# Endangered Black‐faced Spoonbills alter migration across the Yellow Sea due to offshore wind farms

**DOI:** 10.1002/ecy.4485

**Published:** 2024-11-27

**Authors:** Yi‐Chien Lai, Chi‐Yeung Choi, Kisup Lee, In‐Ki Kwon, Chia‐Hsiang Lin, Luke Gibson, Wei‐Yea Chen

**Affiliations:** ^1^ Department of Environmental Science and Engineering Tunghai University Taichung Taiwan; ^2^ Environmental Research Center Duke Kunshan University Kunshan Jiangsu China; ^3^ School of Environmental Science and Engineering, Southern University of Science and Technology Shenzhen China; ^4^ Waterbird Network Korea Seoul Republic of Korea; ^5^ Research Center for Endangered Species National Institute of Ecology Yeongyang Republic of Korea

**Keywords:** barrier effect, East Asian‐Australasian Flyway, green energy infrastructure, migration abandonment, *Platalea minor*, telemetry, waterbirds

The Black‐faced Spoonbill (*Platalea minor*), an endangered and flagship species inhabiting coastal wetlands along the East Asian‐Australasian Flyway (BirdLife International, 2017; Cultural Heritage Administration, 2020), migrates annually between its primary breeding grounds on the west coast of the Korean Peninsula (Kang et al., 2016) and its wintering grounds, predominantly Japan, Chinese mainland, Hong Kong, Taiwan, and Vietnam (Yu et al., 2023). The Yellow Sea crossing, averaging 14.1 h (Appendix S1: Table S1), is perhaps the most challenging part of their migration. The southwest coast of the Yellow Sea, crucial for migratory birds, hosts the world's largest concentration of operational offshore wind farms. In 2021, driven by the government's year‐end deadline for receiving subsidies for offshore wind energy generation, a surge in installations occurred in China. The surge resulted in China possessing nearly half of the world's total offshore wind energy capacity (Global Wind Energy Council, 2023). While the barrier effect, which impedes the natural movement of wildlife, caused by individual offshore wind farms is generally considered marginal for nonmarine bird species compared with seabirds (Fox et al., 2006; Masden et al., 2009; Masden et al., 2010; Pettersson, 2005), the cumulative effects of multiple wind farms may be substantial. Here, we report two cases of GPS‐cellular tracked Black‐faced Spoonbills altering their migration routes during the Yellow Sea crossing after encountering successive offshore wind farms.

In the first case, M03, a male in its hatching year, departed South Korea on its first southward migration at dawn on 2021‐11‐07 (Video S1). After covering a distance of 502.0 km in 10.5 h, it was 65.9 km off the Rudong coast, one of the most important stopover sites for Spoon‐billed Sandpiper in Jiangsu, China (Peng et al., 2017; Yang et al., 2020), when it sequentially entered two offshore wind farms (Figure 1a). M03 flew mainly at the height of the blade rotation zone (see details in Appendix S1). After passing through the two wind farms, it altered its direction and headed north. After dusk, M03 encountered a third wind farm, where it spent 1.0 h circling before departing and reversing its path back to South Korea. It then flew a distance of 376.8 km, landing in the middle of the night on 2021‐11‐08 on the rocky shore of Hatae Island, around 110 km west of the Korean Peninsula. Notably, this trip recorded the second longest continuous flight among all the other 38 Yellow Sea crossing tracks collected in the study, with a duration of 21.9 h (Appendix S1: Table S1). The bird remained stationary for 29.5 h, indicating possible exhaustion, before resuming its journey toward Haenam County at the southern tip of the Korean Peninsula. Following its return, M03 mainly stayed in this region, exploring Gusan Stream and the intertidal flats, and did not attempt further migration. South Korea has a small wintering population of up to 53 Black‐faced Spoonbills on Jeju Island, 85 km south of the mainland (Yu et al., 2023). Although individuals occasionally appear on the mainland during the January census, M03 did not survive the winter and was found dead in Gusan Stream in late December.

**FIGURE 1:**
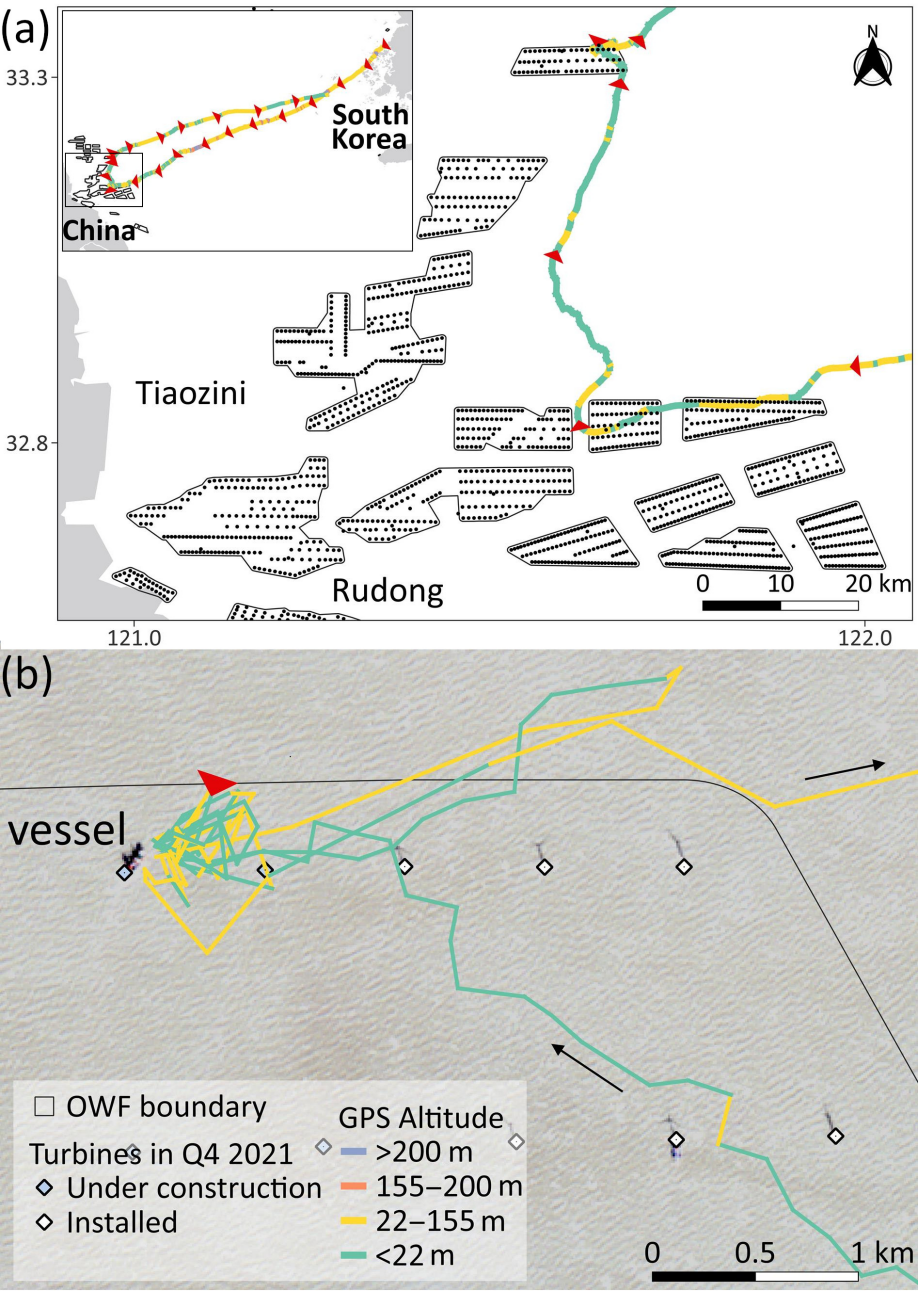
(a) M03 encountered three offshore wind farms (OWFs) before returning to South Korea during its first southward Yellow Sea crossing. The red arrowhead indicates the track's position and direction at the top of each hour. (b) M03 circled beside a turbine under construction in the third wind farm after dusk. Sentinel‐1 satellite images on 2021‐11‐09, 2 days after M03's passage, showed an installation vessel. Satellite images were downloaded through Google Earth Engine. The four GPS altitude intervals are related to the height of the blade rotation zone: <22 m (below it), 22–155 m (within the typical range), 155–200 m (potentially within the range of newer turbines), and >200 m (above it) (see Appendix S1 for detail).

Combined with the wind data, M03 initially took flight in calm easterly winds but encountered a powerful westerly air current exceeding 15 m/s after it headed north toward the third wind farm (Video S1). Satellite images from Sentinel‐1 revealed that the three wind farms were still under construction during M03's passage on 2021‐11‐07, and were all grid‐connected by the end of the year. Based on available images taken on November 4, 9, 14, and 19, a vessel was present on 9th and the 14th, 2 and 7 days after the bird's passage, where M03 circled for an hour in the third wind farm (Figure 1b). On 19th, the turbine was fully installed. Although no image was available for the day of M03's passage, it is plausible that the installation vessel may have been present, potentially attracting or misleading the bird while it faced the strong wind at night.

In the second case, Y70, a male in its third calendar year undertaking its first northward Yellow Sea crossing, encountered offshore wind farms on two attempts before successfully completing its flight to South Korea (Figure 2a). On 2022‐05‐02, Y70 arrived at Tiaozini wetland in the Yellow Sea, the core area of a World Heritage site on the east coast of China, after departing from Taiwan in late April. On May 28, it departed from Tiaozini wetland at dusk and passed through two wind farms (Figure 2b). Despite eventually raising its flight altitude to avoid the blade rotation zone, it returned along a similar route, covering a distance of 76.6 km in 1.4 h. Its second attempt was at dawn on May 31. After flying for 1.2 h, it encountered the first wind farm. It changed direction toward the east to avoid it and flew in a rather tortuous path before entering a second one. It flew at the height of the blade rotation zone and spent 30 min in the second and third wind farms. After exiting, it flew back toward Tiaozini, covering a distance of 164.4 km in over 4 h. It then continued flying along the coast toward the north, revisiting the northernmost point it had reached during its first northward migration the previous year, before returning to Tiaozini by the end of the day. In both attempts, Y70 did not encounter adverse weather conditions, such as unfavorable winds or rainfall. And the offshore wind farms it came across were all operational. Eventually, after postponing its departure for over half a month, Y70 took off in the early morning on June 15 and headed for South Korea, making its departure the last among all the other tracks that migrated back to breeding grounds (Figure 2c).

**FIGURE 2:**
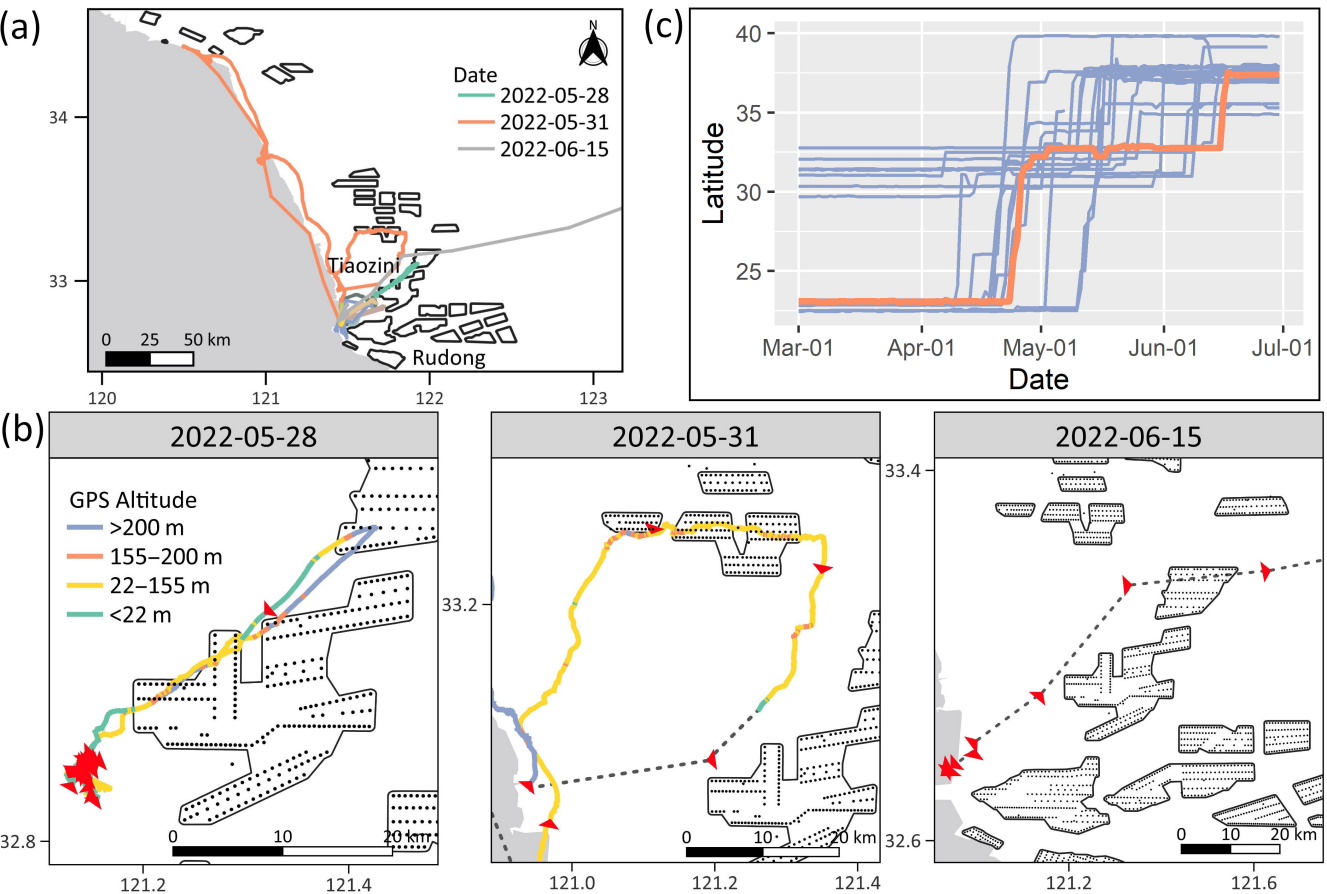
(a) Daily tracks of Y70 from 2022‐05‐28 to 2022‐06‐15. The three highlighted days indicate two migration attempts and one successful track during its northward Yellow Sea crossing. On other days, Y70 stayed around Tiaozini mudflats. (b) Y70 encountered several offshore wind farms during migration attempts on May 28 and 31 before its final departure on June 15. Dashed lines represent segments with reduced positioning frequency to once an hour. (c) Latitudinal movement of the 20 tracks visiting breeding grounds during northward migration, including Y70 in 2022, is highlighted in orange.

Jacobsen et al. (2019) documented that a portion of migrating raptors turned back toward the mainland after avoiding an offshore wind farm in the Baltic Sea, but the observational data did not provide information on their subsequent behavior. Studies on nocturnally migrating passerines showed that reverse migration occurred more frequently among juveniles, lean individuals, and during unfavorable weather conditions when facing a sea crossing (Nilsson & Sjöberg, 2016). In the two reported cases, both inexperienced young birds with normal body conditions passed through multiple wind farms off the coast of Rudong and Tiaozini in southern Jiangsu, a region with a high density of offshore wind farms. Apart from M03, of the eight other southward tracks that approached China through this region, three landed in the wind farms on the intertidal flats, four flew over them, and one passed through at the height of the blade rotation zone (Appendix S1: Figure S1a). Among the four northward tracks departing from this region besides Y70, one flew below the blade rotation zone, two adjusted their height but still spent a considerable amount of time flying at this height, and one landed in a wind farm and resumed migration after an 18‐min break (Appendix S1: Figure S1b). This indicates that individuals passing through the region are impacted by these structures to varying degrees, although further analysis is needed. With a suitable tide, birds might be able to land when encountering offshore wind farms. However, if no landing sites are available, they need sufficient energy reserves to navigate through the barriers created by multiple offshore wind farms.

The trackers cannot record fatal incidents during sea crossings due to a lack of cellular connections. In the two reported cases, encounters with operational or under‐construction wind farms did not result in direct mortalities. The avoidance behavior could reduce collision mortality (Cook et al., 2018), potentially mitigating the negative impact of these installations (Plonczkier & Simms, 2012). However, in areas with high densities of wind farms, the barrier effect of these structures still poses substantial stress, compounding existing migration challenges such as the unfavorable weather encountered by M03. This stress could potentially alter their migration behavior, leading to delays, migration abandonment, or even death out of exhaustion. The fact that M03 eventually died after failing to migrate south indicates that the current impact studies relying on collision mortality estimation may underestimate the overall negative impacts on migratory species, which could lead to mortality following transit rather than direct collisions.

Although offshore wind farms are mainly concentrated in the southwestern part of the Yellow Sea, their rapid expansion is expected across Asia. For instance, continuous development zones are projected for the northwest coast of Taiwan (4C Offshore, n.d.). However, there is a noticeable absence of publications using high‐temporal resolution tracking data to quantify their impact on migratory birds on the flyway. And it remains unclear whether the gaps between different wind farms serve as passages for them. The Black‐faced Spoonbill, a species capable of carrying relatively heavy trackers, presents an excellent subject to examine how migratory birds reliant on the Yellow Sea might respond to the high density of offshore wind farms, while also providing insights for development zones elsewhere. Further research is urgently needed to quantify behavioral responses (Schwemmer et al., 2023) and assess the cumulative impacts on them and other migratory birds along the East Asian‐Australasian Flyway.

## CONFLICT OF INTEREST STATEMENT

The authors declare no conflicts of interest.

## ETHICS STATEMENT

The capture of all individuals was carried out after receiving permission from the Cultural Heritage Administration of Korea.

## Supporting information


Appendix S1.

Video S1.

Video S1 Legend.

## Data Availability

Data (Lai et al., 2024) are available in Figshare at https://doi.org/10.6084/m9.figshare.25398430. The offshore wind trubine dataset has been updated to 2022 based on Zhang et al. (2021) and Hoeser et al. (2022). See Appendix S1 for detailed methods.
