# Supplementary material for: Endangered Black‐faced Spoonbills alter migration across the Yellow Sea due to offshore wind farms
Source: Ecology. 2024 Nov 27;106(1):e4485. doi: 10.1002/ecy.4485 (PMC11733854; doi:10.1002/ecy.4485)
Supplement: Supplementary file 1 — Appendix S1. [file ECY-106-e4485-s001.pdf]

Ecology

Endangered black-faced spoonbills alter migration across the Yellow Sea due to offshore wind farms

Yi-Chien Lai, Chi-Yeung Choi, Kisup Lee, In-Ki Kwon, Chia-Hsiang Lin, Luke Gibson, Wei-Yea Chen

Appendix S1

Bird banding

Post-fledging juveniles were captured using hand nets on their nests on islets in South Korea between June and July. The birds were fitted with a GPS-cellular tracker attached via a Teflon ribbon backpack harness. The devices, weighed between 1.2% and 1.8% of the bird's body mass (1.31 to 2.04 kg), were LEGO 3G EL26 model manufactured by the Druid Technology Co., Ltd. in China. These trackers were set to record position, altitude (height above mean sea level) and instantaneous speed at default intervals of 1 hour. However, during flight (instantaneous speed exceeding 2 m/s) and with sufficient solar battery charge, the data collection frequency could automatically increase to intervals as short as 20 seconds. Data collected was stored in the device and uploaded to a compatible cellular network when available. Sea crossing is the non-stop flight from take-off to landing across the Yellow Sea or the Taiwan Strait (Table S1). A speed threshold of 2.5 m/s was applied to filter flying segments. However, if the speed dropped below 2.5 m/s for no longer than 5 minutes, it is still considered as a non-stop flight, as slower speeds may occur during circling. Instantaneous speed recorded by the tracker was used for segments with GPS intervals under 3 minutes, while the average speed between two GPS points was used for intervals over 3 minutes.

Table S1. Summary of sea-crossing flights of tracked Black-faced Spoonbill collected between mid-2019 and mid-2023. Only tracks with position intervals of 12 minutes or less are included. As M03 did not complete the Yellow Sea crossing, its track is excluded.

Sea	Direction	Duration	Distance	Average Speed	n
Yellow Sea	southward	13.8 ± 3.7 hr (8.7- 21.2 hr)	737.9 ± 166.8 km (526.4- 1261.2 km)	54.6 ± 7.8 km/hr (41.2-69.3 km/hr)	25
Yellow Sea	northward	14.7 ± 3.7 hr (8.9- 23.3 hr)	744.0 ± 128.1 km (553.4- 957.4 km)	52.5 ± 11.5 km/hr (35.0-76.5 km/hr)	13
Taiwan Strait	southward	8.3 ± 2.8 hr (4.8- 12.1 hr)	531.7 ± 241.3 km (287.1- 844.4 km)	63.5 ± 15.5 km/hr (38.7-85.7 km/hr)	8
Taiwan Strait	northward	12.2 ± 3.5 hr (6.8- 17.3 hr)	472.1 ± 113.4 km (314.8- 640.5 km)	40.3 ± 9.1 km/hr (27.3-51.9 km/hr)	10

Values are presented as Mean ± Standard Deviation. Ranges are indicated in brackets.

Offshore wind farm dataset

We used the offshore wind turbine dataset from Hoeser, Feuerstein, and Kuenzer (2022) and updated it from the second quarter (Q2, April to June) of 2021 to Q4 2022 on the Google Earth Engine platform, by using the Zhang et al. (2021) dataset or manually digitizing based on the quarterly median composite images generated with the methods from Hoeser, Feuerstein, and Kuenzer (2022). We identified wind farm boundaries using a 3km threshold, rather than relying on developers' delineations. Turbines that were either constructed or under construction as of Q4 2022 were included, and then any two turbines within 3 km of each other were grouped into the same wind farm.

The heights of the blade rotation zone were calculated by subtracting and adding the length of the turbine blade to the height of the hub. According to the environmental impact assessment reports of offshore wind farms around the Yellow Sea, the height of blade rotation zone spans from 22 to 155 m for most turbines grid-connected by 2020 in China. Newer turbines installed after 2021 have longer blades, extending the height of the blade tip to nearly 200 m. Since we did not obtain the exact height for each turbine, we categorized the birds' flight altitudes into four

intervals to distinguish the degree of influence from the turbines: 22-155 m, representing altitudes within the typical height of blade rotation zone of almost all the turbines; 155-200 m, potentially within the extended rotation zone of fewer newer turbines; and <22 m or >200 m, which fall outside the height range of the blade rotation zone.

Other observed tracks

The birds arriving at and departing from the coast of China through the Tiaozini and Rudong mudflats in southern Jiangsu, a region with a high density of offshore wind farms, were considered to be primarily impacted by the continuous presence of these wind farms. We illustrated these tracks passing through the region between mid-2019 and mid-2023 (Figure S1).

Landing in the wind farms was also one of the behaviors indicating that the birds' migrations were impacted by the installations, although one might argue that landing on intertidal flats is normal for Black-faced Spoonbills. Of the 25 southward tracks across the Yellow Sea, 44% landed on supratidal area, while 56% landed on intertidal zone. However, almost all tracked individuals landed within 1.2 km off the coast, except for the three tracks passing through the southern Jiangsu region, which landed 4.9, 20.8, and 30.8 km off the coast in the wind farms (Figure S1 (a)). While the extensive intertidal areas here could explain this, M60's track showed atypical behavior (marked with * in Figure S1(a)). Unlike the other two tracks that landed at night during low tide and were moved towards the coast by the rising tide the next morning, M60 landed in the morning, 17 minutes after high tide in a possible laver farm (Lu et al. 2019), when the sandbank should have been underwater according to the satellite image. The bird remained there for 54.9 hours before moving to other sandbanks, suggesting exhaustion after a 17.5-hour flight. This indicates the potential challenges and impacts of

offshore wind farms on birds with very tight energy budgets during migration.

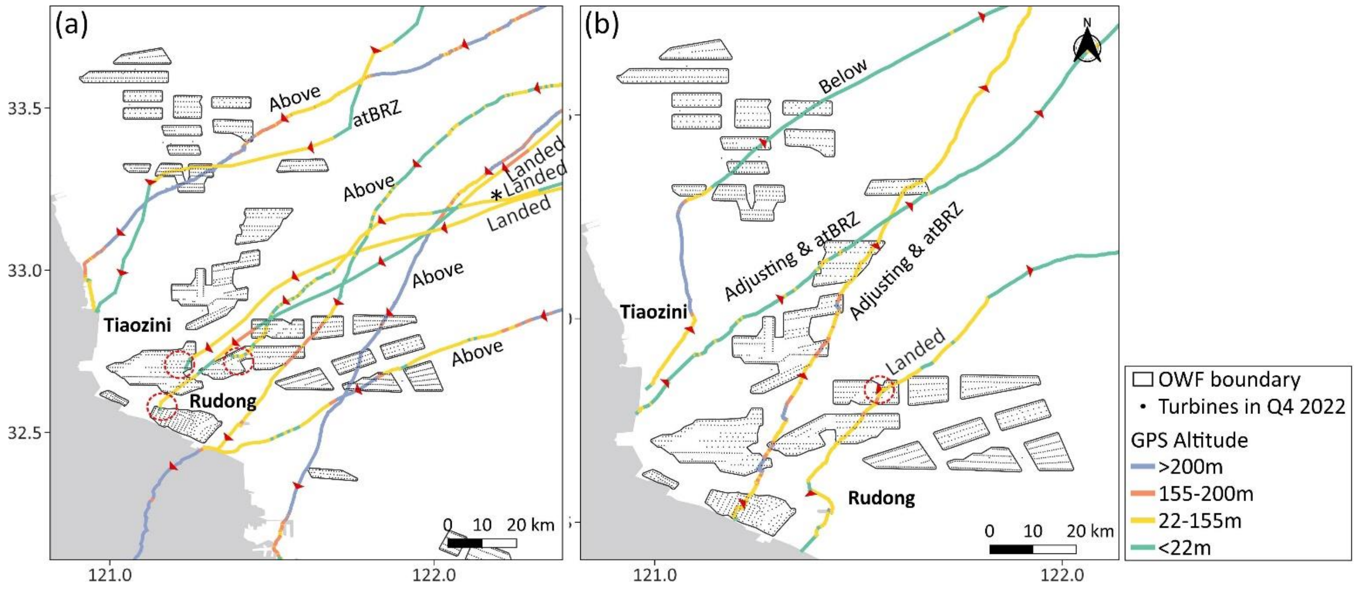

Figure S1. Tracks passing through Tiaozini and Rudong mudflats during (a) southward and (b) northward migration. Tracks that landed in the offshore wind farms have their landing positions circled with red dashed lines. * marked M06's track, which landed 30.8 km off the coast during high tide. The red arrowhead indicates the tracks' position and direction at the top of each hour.

Data analysis

The analysis was conducted in R version 4.2.2 (R Core Team 2022). The "suncalc" package (Thieurmel and Elmarhraoui 2022) was used to obtain the time of "dawn" and "dusk" at specific locations. Spatial analysis was done with the "sf" package (Pebesma 2018). The map was created using the software QGIS version 3.20.0-Odense (QGIS Development Team 2021) with coastline data (OpenStreetMap contributors n.d.) retrieved through FOSSGIS e.V. (n.d.).

References

FOSSGIS e.V. n.d. "Land polygons. Large simplified polygons not split, use for zoom level 0-9." Accessed 02 July, 2023. https://osmdata.openstreetmap.de/data/land-polygons.html.

Hoeser, Thorsten, Stefanie Feuerstein, and Claudia Kuenzer. 2022. "DeepOWT: a global offshore wind turbine data set derived with deep learning from Sentinel-1 data." *Earth System Science Data* 14 (9): 4251-4270. https://dx.doi.org/10.5194/essd-14-4251-2022.

Lu, Wanyun, Jiaqi Sun, Yongxue Liu, Yongchao Liu, and Bingxue Zhao. 2019. "Seasonal and Intra-Annual Patterns of Sedimentary Evolution in Tidal Flats Impacted by Laver Cultivation along the Central Jiangsu Coast, China." *Applied Sciences* 9 (3): 522. https://dx.doi.org/10.3390/app9030522.

OpenStreetMap contributors. n.d. Coastlines.

Pebesma, Edzer. 2018. "Simple Features for R: Standardized Support for Spatial Vector Data." *The R Journal* 10 (1): 439-446. https://doi.org/10.32614/RJ-2018-009.

QGIS Development Team. 2021. "QGIS Geographic Information System" V. 3.20.0-Odense. QGIS Association. http://www.qgis.org.

R Core Team. 2022. "R: A Language and Environment for Statistical Computing" V. 4.2.2. R Foundation for Statistical Computing, Vienna, Austria. https://www.R-project.org/.

Thieurmel, Benoit, and Achraf Elmarhraoui. 2022. "suncalc: Compute Sun Position, Sunlight Phases, Moon Position and Lunar Phase" V. 0.5.1. https://CRAN.R-project.org/package=suncalc.

Zhang, Ting, Bo Tian, Dhritiraj Sengupta, Lei Zhang, and Yali Si. 2021. "Global offshore wind turbine dataset." *Scientific Data* 8 (1). https://dx.doi.org/10.1038/s41597-021-00982-z.
