# Supplementary material for: Endangered Black‐faced Spoonbills alter migration across the Yellow Sea due to offshore wind farms
Source: Ecology. 2024 Nov 27;106(1):e4485. doi: 10.1002/ecy.4485 (PMC11733854; doi:10.1002/ecy.4485)
Supplement: Supplementary file 3 — Video S1 Legend. [file ECY-106-e4485-s003.pdf]

*Ecology*

# Endangered black-faced spoonbills alter migration across the Yellow Sea due to offshore wind farms

Yi-Chien Lai, Chi-Yeung Choi, Kisup Lee, In-Ki Kwon, Chia-Hsiang Lin, Luke Gibson, Wei-Yea Chen

## Video S1 Metadata

M03's southward Yellow Sea crossing track along with accompanying wind patterns. It was created in QGIS using temporal controller (QGIS Development Team, 2021). The wind data comprised the 10m u- and v- components obtained from the "ERA5 hourly data on single levels from 1940 to present" dataset downloaded from the European Centre for Medium-range Weather Forecast (Hersbach et al., 2023).

## References

Hersbach, H., B. Bell, P. Berrisford, G. Biavati, A. Horányi, J. Muñoz Sabater, J. Nicolas, C. Peubey, R. Radu, I. Rozum, D. Schepers, A. Simmons, C. Soci, D. Dee, J-N. Thépaut. 2023. ERA5 hourly data on single levels from 1940 to present. Copernicus Climate Change Service (C3S) Climate Data Store (CDS), https://doi.org/10.24381/cds.adbb2d47 (Accessed on July 18, 2022)

QGIS Development Team. 2021. "QGIS Geographic Information System". V. 3.20.0-Odense) QGIS Association. http://www.qgis.org
